# Understanding Perceptions of Mental Health Practitioners on Care and Treatment Reviews (CTRs)

**DOI:** 10.1192/bjo.2024.107

**Published:** 2024-08-01

**Authors:** Ayomipo Amiola, Verity Chester, Kiran Purandare, Rohit Shankar, Regi Alexander

**Affiliations:** 1 Hertfordshire Partnership University NHS Foundation Trust, Norwich, United Kingdom; 2 RADiANT, Norwich, United Kingdom; 3 The Learning Disabilities Directorate Central and North West London Foundation NHS Trust, London, United Kingdom; 4 Cornwall Partnership NHS Foundation Trust, Plymouth, United Kingdom; 5 Peninsula School of Medicine, University of Plymouth, Plymouth, United Kingdom; 6 University of Hertfordshire, Hatfield, United Kingdom

## Abstract

**Aims:**

NHS England's ‘Transforming Care’ initiative introduced care and treatment reviews (CTRs) for adults with intellectual disabilities and/or autism to avoid inpatient admissions, improve inpatient care quality and support timely discharge. CTRs are completed by an independent panel including an expert by experience, a clinician, and the commissioner. Since 2015, thousands of CTRs have been carried out. In a survey of ID psychiatrists involved in CTRs, many felt that discharge planning was limited by a lack of appropriate community placements. Proposed changes to the Mental Health Act indicate that CTRs should become statutory.

Our aim was to obtain the views of professionals working in intellectual disability services on the proposed reforms to the Mental Health Act and CTRs.

**Methods:**

A mixed methods 34-item questionnaire exploring views of professionals working in ID services (n = 66) on the CTR process, their perception on its usefulness and the proposal to make CTR recommendations statutory. Survey shared with ID MDT professionals working in the UK. Of the respondents, 30% were psychiatrists, 29% psychologists and 21% nurses, with average length of mental health service of 18.2 years. More than 80% work in the NHS and most worked either in inpatient or forensic units.

**Results:**

Although in 80% of CTRs attended, patients have a current risk assessment and management plan, in less than 10% of CTRs attended were people ready for discharge and had a current discharge plan. In terms of CTR actions, 70% of the time, patients were receiving the right care, over 60% of the time, care was person centred, person's health needs are met and 50% of the time key areas of concerns were covered. In less than 40% of CTRs were the person's rights always upheld, family or carers always involved, medications being used appropriately or were there clear, safe and positive approaches to risk. Reasons for delayed discharges included no placement options (68%), no placement profile or community needs assessment (24%), placement funding disputes (23%), no agreed social care responsibility (18%) and no agreed community clinical care responsibility (18%). Only 7% of respondents felt CTRs were always useful, 44% felt they were sometimes useful and 23% often useful. Professionals had mixed views about whether CTRs should become statutory/enforceable (45%) versus those who did not (48%).

**Conclusion:**

This is a survey with a relatively representative sample of MDT professionals involved in CTRs. It gives insight into the typical CTR process, duration, and professionals involved. It summarises the opinions of clinicians towards CTRs and their views on proposed changes.

